# Seasonal abundance of *Eriosoma lanigerum* (Hausmann) (Hemiptera: Aphididae) and its regulation by *Aphelinus mali* (Haldeman) (Hymenoptera: Aphelinidae) from a 3-year survey in apple orchards

**DOI:** 10.1093/jee/toag084

**Published:** 2026-04-07

**Authors:** Alberto Fassio, Matteo Cottura, Francesco Tortorici, Luciana Tavella

**Affiliations:** Department of Agricultural, Forest and Food Sciences, University of Turin, Torino, Italy

**Keywords:** woolly apple aphid, parasitoid, Aphelinidae, density-dependent regulation, sustainable pest management

## Abstract

*Eriosoma lanigerum* has become an increasing threat to apple orchards, partly due to the withdrawal of some insecticides such as neonicotinoids and organophosphates. Native to North America, this aphid completes its life cycle on apple trees, infesting roots, trunks, and branches, and causing yield losses. Among its natural enemies, the specialist parasitoid *Aphelinus mali* plays a key role in biological control and represents a successful case of classical biological control. However, data on the seasonal abundance of *E. lanigerum* and *A. mali*, and in particular on their interactions under temperate European conditions and different management systems, remain limited. Therefore, 3-year surveys were carried out in 5 apple orchards in northwestern Italy to assess the population dynamics of *E. lanigerum* and its regulation by *A. mali* under temperate climatic conditions. Aphid infestations increased in spring, peaked during summer, and declined toward autumn but varied considerably across years, orchards, and management systems. Infestation pressure was generally higher in organic orchards than in IPM orchards, reflecting differences in pesticide use. The abundance of *A. mali* broadly mirrored aphid dynamics, and parasitism rates generally increased after pest peaks, although timing differed among orchards. Analyses indicated that *A. mali* responds strongly to rising host density until a certain infestation threshold is reached, after which its control effect is expressed mainly through increased parasitism rather than higher adult abundance. These findings emphasize the density-dependent regulation exerted by *A. mali* and provide a quantitative basis for integrating parasitoid activity into decision thresholds for sustainable apple management.

## Introduction

The woolly apple aphid, *Eriosoma lanigerum* (Hausmann) (Hemiptera: Aphididae), is one of the main harmful pests of the apple tree *Malus domestica* (Borkhausen) (Rosales: Rosaceae) worldwide. Native to eastern North America, this pest is now widespread in all apple-growing countries. In literature, it is often reported that, in its native area, *E. lanigerum* overwinters on the primary host, the American elm *Ulmus americana* L. (Rosales: Ulmaceae), before migrating in spring and summer to apple trees and other secondary hosts (e.g., *Crataegus* spp., *Sorbus* spp.) (Patch 1913, Baker 1915, Gontijo et al. 2012). However, this finding remains quite controversial. Other sources suggest that the infestation observed on the American elm in northeastern America should be attributed to other aphid species, particularly *Eriosoma herioti* Börner, *Eriosoma crataegi* (Oestlund), and *Eriosoma americanum* (Riley). For this reason, the American elm would not be the primary host of *E. lanigerum*, which instead appears to be closely associated with apple trees (Börner 1932, Jovičić 2024). That said, the issue merits further research.

In areas where apple cultivation is widespread, *E. lanigerum* lives year-round on *M. domestica*, completing an anholocyclic life cycle with multiple generations of wingless, asexual viviparous females (Nicholas et al. 2005). Nonetheless, winged sexuparae may appear in early autumn and, under conditions of overpopulation or long-distance spread, may migrate to other apple trees and give rise to sexual individuals—males and oviparous females (Sandanayaka and Bus 2005). However, eggs laid on apple trees by oviparous females do not hatch (Jovičić 2024). Usually, *E. lanigerum* overwinters as nymphs, sheltering in bark crevices on the trunk and the first root sections (Beers et al. 2010b).

Depending on the infestation level and colony location, *E. lanigerum* can cause minor damage to apple trees or lead to severe production losses and even tree decline. This insect colonizes root systems, branches, and shoots, settling in pruning cuts and inducing gall formation, resulting in reduced plant vigor (Weber and Brown 1988, Brown et al. 1991, Goossens et al. 2011). The main characteristic of *E. lanigerum* is its ability to produce long, white, waxy filaments secreted by wax glands. This wax performs numerous functions, the most important of which is to protect aphids from fungal infections. The wax prevents the aphid from being coated with honeydew, which otherwise favors fungal development (Su et al. 2016). Moreover, in cases of heavy infestations, colonies located near the spurs may emit honeydew onto the fruit surface, providing a substrate for sooty mold (Stokwe and Malan 2016).

Several natural predators help regulate *E. lanigerum* ­populations, including spiders and insects such as coccinellids and carabids (Coleoptera), hoverflies (Diptera), and earwigs (Dermaptera) (Peñalver-Cruz et al. 2020). In its native range, *E. lanigerum* is effectively controlled by the specialized endoparasitoid *Aphelinus mali* (Haldeman) (Hymenoptera: Aphelinidae) (Asante 1997). The introduction of this wasp for *E. lanigerum* control represents one of the most successful cases of classical biological control (Howard 1929). In Italy, *A. mali* was first introduced in 1921 (Del Guercio 1923).

In contrast to its host, *A. mali* reproduces more slowly (5 to 7 generations per year, compared to 10 to 12 of *E. lanigerum*), parasitizing all parthenogenetic stages of the aphid with a predilection for third-instar nymphs and older hosts (Cross et al. 1999, Bangels et al. 2021). The female lays single eggs into the aphid body; the egg hatches in about 3 days, after which a larva emerges and completes development in about 10 to 12 days. Next, the parasitoid pupates inside the host and emerges as an adult by chewing a circular hole on the aphid body (Mueller et al. 1992). *Aphelinus mali* overwinters as a larva or pupa inside the body of the parasitized *E. lanigerum* individual. Diapause generally begins in October and ends between late March and mid-May, depending on the geographic area. In the early stages of the cycle, no external signs of parasitization are visible: the aphid hosting the *A. mali* larva may continue to grow and develop and even give rise to offspring. The clearest sign of successful parasitization is the appearance, within *E. lanigerum* colonies, of unwaxed, black-colored individuals having a circular hole made by the emerged parasitoid.

The introduction of *A. mali* into new areas infested by *E. lanigerum* has yielded mixed results. Excellent control has been achieved in the northwestern United States, Australia, Canada, New Zealand, Chile, and Uruguay, while the containment of *E. lanigerum* has not always been successful in Europe and parts of Asia (AliNiazee and Croft 1999). Differences in parasitism rates observed in different geographic areas could be partly explained by the fact that the parasitoid is favored by milder temperatures and disadvantaged by cooler ones (Asante and Danthanarayana 1992, Monteiro et al. 2004). Moreover, the presence and abundance of *E. lanigerum*, and consequently the damage to apple production, have increased in apple orchards in recent years, probably due to increasing restrictions and the ­withdrawal of active ingredients worldwide, the permanent side effects of pesticides on natural enemies, the increased survival rate during winter, and the reduced use of resistant rootstocks (Penman and Chapman 1980, Beers et al. 2010a).

The interaction between *E. lanigerum* and *A. mali* has been studied for many years. Although *E. lanigerum* and its natural enemies have been investigated in parts of continental Europe (Helsen et al. 2004, Markó et al. 2008, Happe et al. 2018), field data specifically on *A. mali* remain limited (Evenhuis 1958, Bonnemaison 1965), particularly regarding its numerical response under field conditions. A better understanding of these dynamics is crucial for integrated pest management (IPM) because it shows the host densities at which biological control becomes most effective. Most existing data come from laboratory trials or from related *Aphelinus* species (Frewin et al. 2010, Su et al. 2018, Miksanek and Heimpel 2020), leaving a gap in applied field data for *A. mali*. In continental Europe, organic and IPM apple orchards have rarely been compared, even though management practices strongly affect both aphid pressure and parasitoid activity. Understanding these dynamics is essential for improving pest control, as it helps to define when natural enemies can suppress aphids and when chemical interventions are actually necessary. Therefore, 3-year field surveys were carried out in 5 apple orchards in northwestern Italy, encompassing both organic and IPM systems. Our aims were to (i) describe the seasonal dynamics of *E. lanigerum* and *A. mali*, (ii) quantify the response of the parasitoid to host abundance as a function of density, and (iii) identify the infestation thresholds triggering effective biological control. By combining multiyear field data with segmented regression analysis, this study provides quantitative evidence to support decision thresholds for the conservation and integration of *A. mali* into sustainable apple management strategies.

## Materials and Methods

### Surveyed Orchards

Field surveys were carried out in 5 apple orchards located in Piedmont (NW Italy) over a 3-year period. Specifically, monitoring was performed in three apple orchards (AO1, AO2, and AO3) from 2021 to 2023 and in two further orchards (AO4 and AO5) during 2022 and 2023. The location and main characteristics of the 5 apple orchards are provided in Table 1. In IPM orchards, aphid control relied mainly on active ingredients considered to be relatively more selective (e.g., flonicamid, pirimicarb, pyriproxyfen, and sulfoxaflor), applied in accordance with the Piedmont Regional Guidelines for Integrated Production in force for each year of the study (Regione Piemonte 2021, 2022, 2023). In organic orchards, botanical insecticides (primarily azadirachtin), horticultural oils, potassium salts of fatty acids, and occasionally washing of colonies with water were applied. An apple orchard (AO2) switched from organic management (2021–2022) to IPM (2023), following the grower’s decision to adopt a more effective strategy in response to recurring severe pest infestations. Further details on orchard characteristics and pest management practices, including cultivars, rootstocks, the type, timing, and frequency of insecticide applications, and alternative control measures adopted in each orchard and year, are available in Supplementary Information, Table S1.

**Table 1.** Description of surveyed apple orchards in Piedmont, Italy, monitored during 2021–2023

Id	Site	Coordinates	Management	2021	2022	2023
**AO1**	Campiglione Fenile (TO)	44.810253°N, 7.310948°E	Organic	×	×	×
**AO2**	Saluzzo (CN)	44.623639°N, 7.471417°E	Organic (2021–2022), IPM (2023)	×	×	×
**AO3**	Verzuolo (CN)	44.593528°N, 7.520028°E	IPM	×	×	×
**AO4**	Savigliano (CN)	44.605889°N, 7.622389°E	Organic		×	×
**AO5**	Revello (CN)	44.640333°N, 7.386889°E	Organic		×	×

For each orchard, site locality, geographic coordinates, and management type (organic or integrated pest management, IPM) are given. The symbol “×” denotes years in which monitoring of *Eriosoma lanigerum* and *Aphelinus mali* was conducted.

### Aerial Infestation of *E. lanigerum*

To assess the level and seasonal dynamics of *E. lanigerum* infestations on the aerial parts of apple trees, standardized monitoring by visual inspection was carried out in all surveyed orchards. At each site, 100 current-year shoots (maximum length: 0.5 m) were selected from trees located along a diagonal transect within the orchard to ensure representative sampling across the field. Each shoot was carefully examined, and all active colonies of *E. lanigerum* were recorded and classified based on their size as follows. Each detected colony was assigned to one of three severity classes based on the length of the shoot portion covered by the colony, namely:

- Class I: colonies smaller than 5 mm;
- Class II: colonies between 5 and 10 mm;
- Class III: colonies between 10 and 20 mm.

The aerial infestation for each shoot was calculated as a weighted sum, obtained by multiplying the number of colonies in each severity class by the corresponding class weight (ie, 1 for Class I, 2 for Class II, and 3 for Class III) and summing the products. The average aerial infestation per orchard and sampling date was then computed based on the 100 surveyed shoots and used in the statistical analyses.

Monitoring was performed at fortnightly intervals from April to October each year, thereby covering the main period of colony development on the aerial parts of the tree.

### Abundance of *A. mali*

To monitor the population dynamics of *A. mali*, one yellow sticky trap (dimensions: 0.25 × 0.16 m; Biogard, Grassobbio, Italy) was placed at the center of each orchard, approximately 2 m above ground level. This trap type was selected because it is reported to be the most attractive to the parasitoid (Beers 2012). From March to October each year, traps were exposed and replaced every 2 weeks or immediately after any chemical treatments, to assess the potential impact of pesticide applications on *A. mali* populations. After removal, traps were ­individually wrapped in plastic film and transported to the laboratory, where they were stored at temperatures below 5 °C until analysis.

In the laboratory, *A. mali* adults were counted under a stereomicroscope using an overlapping grid divided into 8 cm^2^ cells. To estimate the presence and abundance of the parasitoid, 10 cells per trap (5 on each side) were selected along the diagonal of the trap. Based on the counts, the daily mean number of individuals captured per cm^2^ for each trap was calculated.

### Parasitism Rate

The parasitism rate of *A. mali* on *E. lanigerum* was assessed by weekly sampling from May to October each year. Ten colonies were randomly collected from infested shoots located approximately 1.5 m above ground within each orchard. Each colony was placed into an individual test tube (30 mm diameter × 100 mm length), sealed with an absorbent cotton plug, and stored at 5 ± 1 °C until analysis. Using a stereomicroscope, the number of parasitized aphids was recorded. Parasitized individuals were identified by the lack of wax coating, their black coloration, and especially the presence of a circular hole caused by the emergence of adult parasitoids. The parasitism rate was calculated as the proportion of parasitized aphids out of the total number of individuals within each colony.

### Statistical Analysis

All statistical analyses were performed using R software v. 4.4.2 (R Core Team 2025). Infestation severity was quantified using the weighted sum described above. For each orchard, sampling date, and year, the mean weighted sum across the 100 surveyed shoots was used as the infestation metric in all subsequent analyses. To examine seasonal patterns of infestation, the daily aggregated weighted sum was plotted against the day of the year and smoothed (method LOESS, span = 0.4) in the ggplot2 package v. 4.0.1 (Wickham 2016). To compare infestation severity between orchards and management systems, a linear model (lm function) in the base Stats package (v. 4.5.1; R Core Team 2025) was fitted with the weighted sum of colonies as the response variable and orchard × year as fixed factors. Estimated marginal means were obtained using the emmeans package (v. 2.0.0; Lenth and Piaskowski 2025), followed by pairwise contrasts within each year. The *P*-values were adjusted using the Šidák correction (García-Pérez 2023). Homogeneous groups were identified using the compact letter display from the multcompView package (v. 0.1.10; Graves et al. 2024).

To analyze the seasonal dynamics of both *E. lanigerum* and *A. mali*, infestation level, parasitism rate, and *A. mali* capture by traps were harmonized on a unified daily temporal scale. Daily mean infestation and parasitism values were computed on the original sampling dates. Since the sticky trap records catches over multiday exposure intervals, daily parasitoid densities were obtained through linear interpolation between each pair of consecutive trap inspection dates. For every orchard and year, the minimum and maximum sampling dates across all datasets were identified, and a complete daily date sequence was generated. All variables were then merged onto this continuous timeline.

To enable comparability among variables with different units and scales, the three daily variables (infestation level, parasitism rate, and parasitoid density by traps) were normalized to the range 0 to 1 within each orchard-year combination using min-max scaling, the rescale() function from the scales package, v. 1.4.0 (Wickham et al. 2025), as recommended for environmental indicators in Mazziotta and Pareto (2022). Seasonal patterns were analyzed by associating each date with its calendar week number and by smoothing the normalized daily values using Generalized Additive Models (GAMs) implemented in the mgcv package (v. 1.9-1; Wood 2017). Weekly dynamics therefore represent smoothed trends of normalized daily values plotted as a function of calendar weeks.

To investigate whether *A. mali* density responded nonlinearly to *E. lanigerum* infestation, segmented (break-point) regression models were fitted using the segmented package (v. 2.1-4) (Muggeo 2016, 2017). Prior to segmentation, a simple linear model was fitted with parasitoid density (interpolated daily trap density) as the response and unscaled daily infestation severity (mean weighted sum) as the predictor. The segmented model estimated the breakpoint and the slopes of the two linear segments. The analysis was conducted separately for organic and IPM orchards, using the management system to subset the data. This approach enabled us to identify changes in the relationship between infestation and parasitoid density and to estimate the infestation level at which such a change occurred.

Finally, to assess whether the parasitism increased after infestation levels exceeded the estimated threshold, a separate analysis was conducted on organic orchards. The threshold corresponded to the weighted infestation measure (mean weighted sum of colony-size classes) estimated by the ­segmented regression model and is hereafter referred to as the infestation threshold. For each orchard, the earliest date on which the infestation exceeded the threshold value was identified. Sampling points were then classified as occurring “before” or “after” that date. Only records with non-missing parasitism data were retained. For each period, the mean and standard deviation of the parasitism were calculated. Because parasitism data did not meet normality assumptions, differences between the two periods were tested using the Wilcoxon rank-sum test (R Stats Package, v. 4.5.1), and results were visualized with a boxplot.

## Results

### Aerial Infestation of *E. lanigerum*

**Fig. 1.**
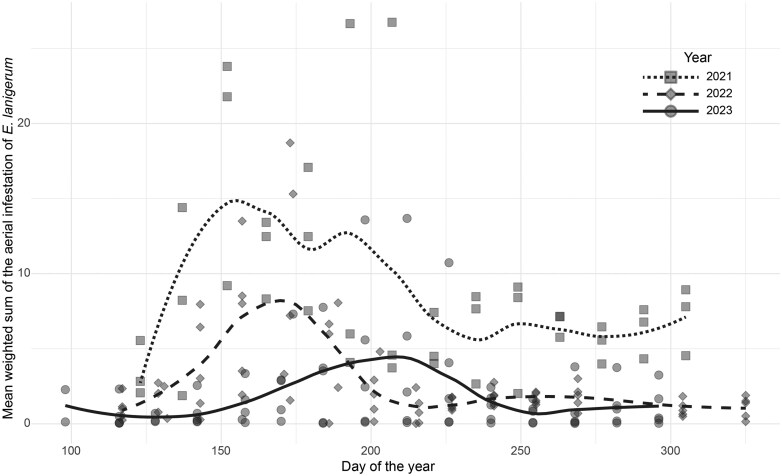
Seasonal patterns of *Eriosoma lanigerum* infestation across the three monitoring years. For each shoot, infestation severity was quantified as a weighted sum, obtained by multiplying the number of colonies in each severity class by its class weight (I, II, or III) and summing the products. For each orchard, sampling date, and year, the mean weighted sum across the 100 surveyed shoots was used as the infestation metric. Daily aggregated values are plotted against the day of the year. Individual observations are shown as grey symbols (2021: squares; 2022: diamonds; 2023: circles), whereas year-specific seasonal trends are represented by LOESS curves (2021: dotted line; 2022: dashed line; 2023: solid line; span = 0.4).

Seasonal monitoring of *E. lanigerum* infestations in the 5 orchards surveyed for 3 consecutive years (2021–2023) revealed distinct and consistent temporal dynamics in both colony development and infestation severity (Fig. 1). Aerial infestation, which incorporates the distribution of colony size, exhibited a similar seasonal pattern across all years. During spring, aerial infestation gradually increased, reaching maximum levels in summer (between the 180^th^ and 220^th^ days of the year). Subsequently, it decreased toward autumn. In particular, infestation pressure was considerably higher in 2021 and 2022, with pronounced peaks in mid-summer. Furthermore, a clear shift in colony composition was observed over time (data not shown), initially dominated by small colonies (Class I), but increasingly characterized by medium and large colonies (Classes II and III) as the season progressed. This pattern suggested rapid colony growth and increased pest pressure during the summer months. In contrast, the 2023 season was characterized by lower aerial infestation values, a more stable seasonal trend, and a reduced occurrence of larger colonies, indicating limited population development. These interannual differences in infestation severity and colony structure highlighted substantial variability in population dynamics between years, likely attributable to a combination of environmental conditions and other biotic factors.

**Fig. 2.**
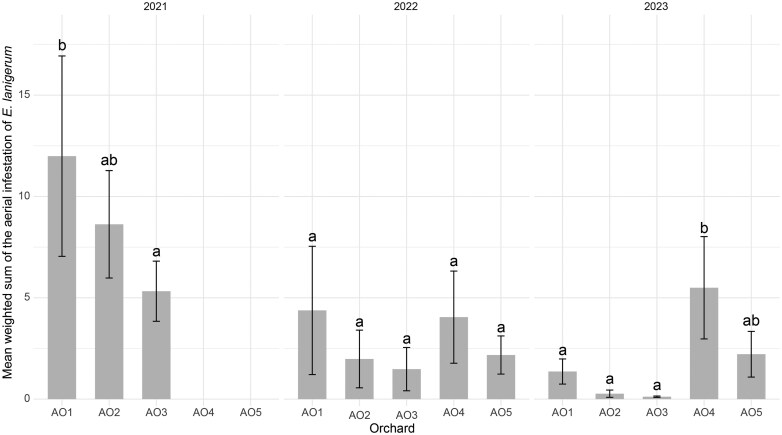
Mean aerial infestation severity of *Eriosoma lanigerum* (± SE) across surveyed apple orchards (AO1–AO5) in each growing season (2021–2023). For each orchard and sampling date, infestation severity was quantified as a weighted sum, obtained by multiplying the number of colonies in each severity class (I–III) by its class weight and summing the resulting values across 100 shoots. Bars represent the mean weighted sum per orchard and year. Different letters indicate statistically significant differences among orchards within each year, based on estimated marginal means with Šidák-adjusted pairwise comparisons (linear model with orchard × year interaction, *P *< 0.05).

A comparison of *E. lanigerum* infestation levels among the 5 monitored orchards (AO1–AO5) was conducted for each growing season based on the mean aerial infestation per sampling date (Fig. 2). The linear model revealed significant effects of orchard [*F*(4, 169) = 6.76; *P *< 0.001], year [*F*(2, 169) = 50.49; *P *< 0.001], and their interaction [*F*(6, 169) = 2.18; *P *= 0.048]. In 2021, infestation severity varied clearly among orchards. AO1 exhibited the highest mean value (11.99), significantly greater than in AO3 (5.32; *t* = 4.70; df = 169; *P *< 0.001), whereas AO2 (8.63) was intermediate and not statistically different from AO1 or AO3. In 2022, infestation pressure, ranging from 4.38 in AO1 to 1.48 in AO3, was generally lower and more uniform with no significant differences among orchards (*P *> 0.05). In 2023, infestation varied again among orchards: AO4 recorded the highest mean value (5.50), significantly exceeding AO1 (*t* = -2.92; df = 169; *P *= 0.0397), AO2 (*t* = -3.69; df = 169; *P *= 0.003), and AO3 (*t* = -3.79; df = 169; *P *= 0.0021). AO5 showed intermediate infestation (2.22) and did not differ significantly from other orchards (*P *> 0.05).

The comparison of *E. lanigerum* infestation levels between organic and IPM apple orchards revealed clear differences between the two management systems. The linear model indicated a significant effect of the management system [*F*(1, 180) = 14.53; *P *< 0.001]. On average, aerial infestation was significantly higher in organic orchards, with a mean estimated infestation level of 4.70 (± 0.42), while IPM orchards showed a considerably lower mean value of 1.80 (± 0.63). The estimated difference between the two systems was 2.90, and this contrast was statistically significant (*t* = 3.81; df = 180; *P* = 0.0002). The nonoverlapping confidence intervals and the grouping of means into distinct statistical classes confirm that *E. lanigerum* pressure was consistently more present under organic management.

### Seasonal Dynamics of *E. lanigerum* and *A. mali*, and Parasitism Rate

**Fig. 3.**
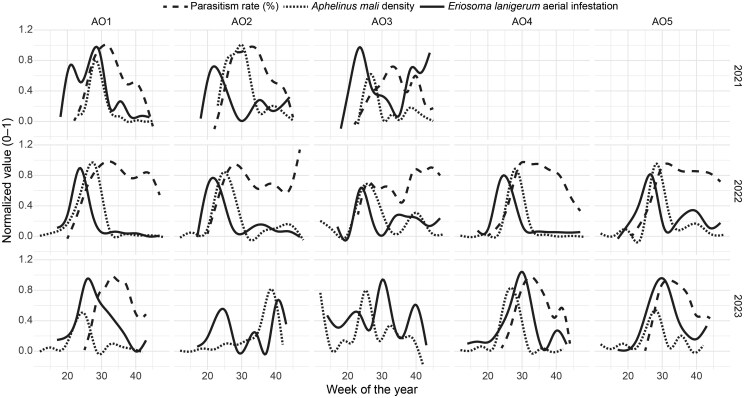
Seasonal dynamics of normalized *Eriosoma lanigerum* infestation, *Aphelinus mali* parasitoid density from sticky trap captures, and parasitism rate across 5 surveyed orchards (AO1–AO5) for the years 2021–2023. Daily mean infestation and parasitism values were calculated at the original sampling dates, while daily parasitoid density was estimated by linear interpolation between consecutive trap inspection dates. For each orchard-year, the minimum and maximum sampling dates were identified, and all variables were harmonized onto a unified daily temporal scale. Variables were then normalized to the range 0 to 1 using min-max scaling to enable direct comparability. Smoothed seasonal patterns were visualized by associating each date with its calendar week and applying generalized additive models to the normalized daily values. Weekly curves represent smoothed trends in pest and parasitoid population metrics, plotted per orchard and year as a function of calendar weeks.

The seasonal trends (rescaled to 0 to 1) of the aerial infestation, *A. mali* abundance, and parasitism rate in the 5 apple orchards monitored over the 3 years (2021–2023) are shown in Fig. 3. Over the 3-year period and across the 5 orchards, all samples exhibited distinct seasonal peaks, generally occurring between weeks 20 and 40. *E. lanigerum* infestations (solid line, Fig. 3) showed one or two distinct peaks per year, suggesting both unimodal (e.g., AO1 and AO4 in 2022, AO1 and AO5 in 2023) and bimodal (e.g., AO1 and AO3 in 2021, AO2 in 2023) seasonal infestation patterns depending on sites and years. The abundance of *A. mali* assessed by yellow sticky traps (dotted line, Fig. 3) tended to follow a seasonal pattern broadly overlapping with pest infestation, suggesting a general correspondence between host and parasitoid dynamics. The peak in parasitoid abundance generally coincided with or followed aphid population peaks, possibly reflecting density-dependent regulation or delayed responses. In 2023, in AO3 and AO4 only, the peak of *A. mali* abundance preceded the infestation peak by a few weeks. The parasitism rate (dashed line, Fig. 3) often increased after infestation peaks, with varying degrees of synchrony depending on the orchard and year. In AO2 and AO3, parasitism rate data were not available due to the phytosanitary treatments applied in these two apple orchards. AO1 showed relatively consistent synchrony between aphid infestation, parasitoid abundance, and parasitism over the 3 years. AO3 exhibited complex infestation dynamics with a strong late-season second peak in 2023, while parasitoid abundance remained relatively constant across years. In AO5, high parasitism rates were observed coinciding, especially in 2023, with declining aphid infestation, potentially indicating effective control.

### Host-Parasitoid Relationship and Numerical Response

**Fig. 4.**
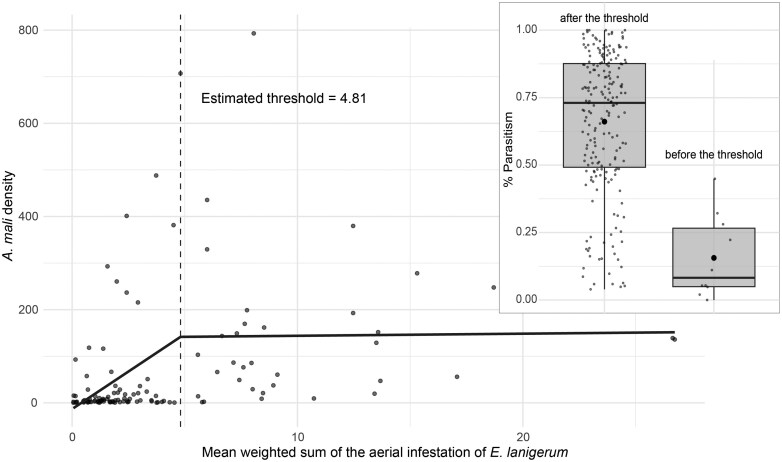
Threshold response of *Aphelinus mali* to *Eriosoma lanigerum* infestation in organic apple orchards. (A) Segmented regression model showing the relationship between infestation severity (expressed as the weighted sum of colony classes) and *A. mali* density. The estimated breakpoint (infestation threshold) at 4.81 indicates a significant change in slope, consistent with a nonlinear density-dependent response of the parasitoid. (B) Parasitism rates before and after the infestation threshold, illustrating the marked increase in parasitism once the threshold is exceeded (Wilcoxon test, *P *< 0.001).

The relationship between *E. lanigerum* infestation levels and the response of its parasitoid *A. mali* in organic orchards is illustrated in Fig. 4. The graph on the left displays a segmented (threshold) regression model describing the relationship between the infestation level of *E. lanigerum* (*x*-axis: aerial infestation) and the interpolated abundance of its parasitoid *A. mali* (*y*-axis) in organic apple orchards. The segmented model (*R*^2^ = 0.160; residual SE = 128.1) (black solid line, Fig. 4) identifies a critical infestation threshold at 4.81 ± 1.56 SE (95% CI = 1.75–7.87) above which the abundance of *A. mali*, assessed using yellow sticky traps, did not increase substantially, suggesting a saturation in the parasitoid response. Below this breakpoint, parasitoid captures increased rapidly with host density (slope = 32.19 ± 12.05 SE; *t *= 2.67, *P *= 0.009), whereas beyond the threshold the slope approached zero (change = -31.74 ± 12.72 SE; *t *=* -*2.50), indicating that adult abundance reached a plateau consistent with a density-dependent but asymptotic response. The graph on the right further explores the effect of surpassing this threshold by comparing parasitism rate before and after the threshold is exceeded. The boxplot shows a significant increase in parasitism after the infestation exceeded the estimated threshold, with a marked increase of median parasitism and a greater concentration of high values compared to the lower and more variable parasitism observed before the threshold. Before the threshold, mean parasitism was 0.156 ± 0.153 SD (*n *= 10), while after the threshold it rose to 0.661 ± 0.260 SD (*n *= 207); the difference was highly significant (Wilcoxon rank-sum test, W = 1940; *P *< 0.001). Together, these results suggest that in organic orchards, *A. mali* is more effective in regulating *E. lanigerum* populations once a critical level of infestation is reached. This supports the hypothesis that a minimum host density is required to trigger a strong parasitoid response, both in terms of abundance and biological control function.

## Discussion

Across sites and years, *E. lanigerum* aerial infestation increased through spring, peaked in mid-summer, and declined into autumn, with a shift from predominantly small to medium/large colonies. This seasonal pattern corresponds to what has been observed in multi-year surveys in commercial orchards, where the movement of the earliest nymphal stages, also known as crawlers, up tree trunks focuses from late spring to mid-summer (Brown and Schmitt 1994, Beers et al. 2010a, 2010b, Lordan et al. 2015), then decreases as plant tissues harden and mortality increases. In Mediterranean orchards, crawlers also move laterally within the canopy during the summer, with colonies in the canopy and roots jointly driving reinfestation (Lordan et al. 2015). Typical establishment points include pruning wounds, bark crevices, and young shoots; moreover, aphid mortality differs according to their position on the plant, with distinct patterns between aerial parts and roots (Jovičić 2024). Over the year, infestation levels varied, with higher and lower pressure in 2021–2022 and in 2023, respectively. Such variability is typical where weather modulates development and survival: warmer springs favor canopy build-up, whereas hot spells and episodic rainfall can suppress aphid performance or disrupt their settlement (Beers et al. 2010a, Lordan et al. 2015). In Piedmont, the Regional Environmental Protection Agency (ARPA Piemonte 2025 Sept 19) ranked 2023 as the second warmest year on record since 1958 (mean ≈ 11.2 °C, +1.3 °C vs 1991–2020) and documented episodes of late summer heat, providing a clear climatic influence for the reduced infestation observed that year. Heat waves and drought are known to depress aphid fitness and can push *E. lanigerum* toward its upper thermal limits (Leybourne et al. 2021), and this could explain the lower aerial infestation recorded in 2023. However, since the experimental design was not structured to quantitatively assess climate effects within each orchard, further studies integrating site-specific weather monitoring would help to better disentangle the climate-infestation relationship.

The differences in *E. lanigerum* pressure across orchards likely reflect a combination of factors such as plant genetics, local conditions, crop management, and the natural enemy community. In our case, orchards AO1 and AO4 were planted with the cultivar “Gala,” which is generally reported as susceptible to *E. lanigerum*, explaining their consistently higher infestation (Abu-Romman and Ateyyat 2014). On the contrary, the orchard AO2, planted with the cultivar “Golden Delicious,” which in a previous study showed very low susceptibility to *E. lanigerum* (Ateyyat and Al-Antary 2009), had generally lower infestation than the other orchards. It is also known that orchards with rootstocks carrying major resistance genes (*Er1*, *Er2*, and *Er3*) tend to show lower *E. lanigerum* infestation than those with susceptible rootstocks (Sandanayaka et al. 2003, Bus et al. 2008). However, all the orchards in this study had trees grafted onto rootstock M9T337, widely described as susceptible to *E. lanigerum*; thus the differences in infestation levels cannot be attributed to the rootstock. In 2023, the most infested orchard was AO4, probably because, being one of the oldest plantations, the trees had numerous wounds and cankers on the trunk and main branches, which favor the overwintering and development of *E. lanigerum* (Weber and Brown 1988, Brown et al. 1991, Asante et al. 1993, Asante 1994, Beers et al. 2010a). In addition, again in this orchard in 2023, the peak of *A. mali* adults occurred before the peak of *E. lanigerum* infestations, and this advance may have limited its effectiveness in controlling the pest, as aphid infestations were still relatively low. This temporal mismatch between parasitoid activity and pest increase highlights the importance of synchrony for effective biological control. Aligning *A. mali* emergence with early aphid build-up should therefore be a management priority and can be supported through degree-day modeling or conservation strategies favoring early parasitoid generations.

Natural enemies add another layer: indeed, the activity of the specialist parasitoid *A. mali* and predator communities varies across orchards mainly due to crop management and context. Although most insecticides used against aphids (e.g., flonicamid, pirimicarb, and pyriproxyfen) in IPM orchards are considered relatively selective and less harmful to parasitoids (Desneux et al. 2007), insecticides approved for organic farming, such as azadirachtin, have been shown to be more compatible with the conservation of natural enemies, including *A. mali*, than broad-spectrum insecticides (Schmutterer 1990). In fact, differences in selectivity contributed to the observed variation in parasitism rate of *A. mali* between different management systems, especially when insecticides are applied during periods of high parasitoid activity (Desneux et al. 2007). It is known that field programs that minimize disruptive chemical interventions have achieved strong pest suppression by *A. mali* (Shaw and Walker 1996, Nicholas et al. 2005, Romero et al. 2025). Concerning predators, earwigs can materially suppress *E. lanigerum*, but their benefits depend on habitat and may emerge only after one or more seasons (Happe et al. 2018, Alins et al. 2023). Broad surveys also show meaningful spatial differences in natural enemy presence across orchards, consistent with variable top-down pressure (Gontijo et al. 2012). Finally, *E. lanigerum* is intrinsically patchy at multiple spatial scales, so even similar orchards can develop persistent hotspots that enlarge between orchard contrasts (Alspach and Bus 1999).

Across this study, aerial aphid infestation tended to be higher in organic orchards than in IPM orchards, as observed in AO3 (for all 3 years) and in AO2 in 2023, where treatments were shown to control infestation satisfactorily. This contrast is best explained by direct chemical suppression of *E. lanigerum* in conventional programs: field evaluations and extension guidance show that appropriately chosen and timed insecticides can effectively reduce the aphid (Jovičić 2024). Overall, evidence indicates that conventional synthetic products tend to achieve higher pest control efficacy compared to organic formulations (Bapfubusa Niyibizi et al. 2025). For AO2, this is what prompted the grower to switch from organic management to IPM after years of recurring severe infestations of *E. lanigerum*. However, this change in management must be taken into account when interpreting infestation trends, and interannual patterns in AO2 must therefore be interpreted with caution. Furthermore, field trials demonstrate that when broad-spectrum pesticides are minimized and parasitoids are conserved, *E. lanigerum* can be contained below damaging levels (Nicholas et al. 2005, Wearing et al. 2010). This is even more important considering the ongoing review and withdrawal of insecticide molecules in Europe, which requires alternative solutions for pest control.

Weekly trends often showed overlapping (sometimes lagged) peaks between aerial infestation, *A. mali* abundance, and parasitism, as expected when a specialist parasitoid tracks its host under relatively undisturbed conditions (Wearing et al. 2010, Gontijo et al. 2012). A modest spring lag in parasitoid pressure is biologically reasonable: *A. mali* generally requires warmer conditions and a greater degree-day accumulation to complete development; thus, its buildup can trail that of *E. lanigerum* early in the season, before synchronizing later (Asante and Danthanarayana 1992, Su et al. 2018). The sampling method used also matters when interpreting *A. mali* activity: adult captures on sticky traps are influenced by trap placement and orientation; therefore, trap data can differ from true parasitism detected on shoots (Beers 2012). Beyond climatic factors and sampling methods, the structure of *E. lanigerum* colonies is important: when colonies merge and the wax layer increases, the internal aphids located deeper remain protected, and *A. mali* mainly attacks the edges. As a result, even if the number of aphids increases, the percentage of parasitization does not grow further and tends to stabilize toward the end of the season (Mueller et al. 1992). Taken together, these mechanisms explain why some orchards in some years show good host-parasitoid synchrony while others exhibit short lags or dampened parasitism despite similar host densities (Wearing et al. 2010, Gontijo et al. 2012). In some orchards (e.g., AO1 and AO3 in 2021, AO2 in 2023), infestation dynamics exhibited a bimodal seasonal pattern, with a second late-season peak. *E. lanigerum* populations, which persist on roots and trunks throughout the season, can act as a reservoir for renewed canopy infestation through crawler movement (Castellari 1967, Heunis and Pringle 2006, Lordan et al. 2015). After partial suppression of aerial colonies due to climatic stress, natural enemy activity, or plant-tissue hardening, a new upward migration from root colonies may contribute to a second peak later in the season. Additionally, high population density may promote the production of winged forms, facilitating short-distance dispersal and recolonization within or among trees (Sandanayaka and Bus 2005). However, these winged forms do not seem to produce offspring on apple trees (Jovičić 2024). The response of *A. mali* to the second peak was not always similar to that observed during the first infestation peak. In several cases, parasitoid density, as detected by traps, remained relatively stable, whereas the parasitism rate appeared to increase after the rise of aphid populations. This suggests that in late season, aphid regulation by parasitoids may be expressed more through sustained parasitism within colonies rather than through a further increase in adult abundance, possibly due to constraints linked to colony structure and host accessibility (Mueller et al. 1992). From a management perspective, these observations indicate that control decisions based solely on aphid density may overestimate risk. Including concurrent parasitism rates provides a more realistic assessment of pest pressure and could reduce unnecessary pesticide use. Decision thresholds that integrate both host density and parasitoid activity are therefore more reliable indicators for intervention.

This study revealed a segmented relationship, characterized by an increase followed by a plateau, between aerial infestation of *E. lanigerum* and the response of its parasitoid *A. mali* in organic orchards. Our analysis identified a critical infestation threshold (∼ 4.81), above which *A. mali* abundance no longer increased substantially, but the parasitism rate rose significantly. This indicates that *A. mali* exerts its strongest regulatory effect once a minimum host density is surpassed, consistent with density-dependent but slowing parasitoid dynamics.

Field investigations and colony-structure analyses provide an explanation for this pattern: as host density increases, the number of parasitized aphids rises, but the proportion of parasitized aphids tends to decline in large mixed colonies because aphids deeper within the colony are better protected by wax and their position, producing apparent saturation at high densities (Mueller et al. 1992, Su et al. 2016). Laboratory and comparative studies further support a Type II functional response for *Aphelinus* species, including *A. mali*, characterized by increasing attacks at low to moderate host densities followed by handling-time-limited saturation. Specifically for *A. mali*, under laboratory conditions, parasitism of *E. lanigerum* followed Holling’s disc equation and fit a Type II model, with attack rates increasing asymptotically and becoming constrained by handling time at higher host densities (Su et al. 2018). Similar density-dependent but saturating responses were reported for congeneric species such as *Aphelinus certus* Yasnosh (Hymenoptera: Aphelinidae), where parasitism increased with aphid density but reached a plateau as host-processing time limited further gains (Frewin et al. 2010). Furthermore, field assessments also indicate that the achieved population suppression may vary across seasons and ecological contexts, suggesting that functional constraints interact with environmental conditions in orchard systems (Miksanek and Heimpel 2020).

Under organic farming systems, these threshold-like dynamics are particularly evident. Our results, which showed a marked increase in parasitism rate once infestation levels exceeded the estimated threshold, are consistent with previous reports of high seasonal parasitism and effective *E. lanigerum* suppression in orchards where chemical treatments are avoided. These findings reinforce the importance of minimizing disturbance in organic apple production. In particular, interventions should be timed to preserve *A. mali* activity (Bangels et al. 2021). This aligns with a key operational principle of IPM: interventions should only occur when the natural control potential is insufficient. Integrating the threshold identified here with phenology-based models could guide such selective decisions, improving economic and environmental sustainability.

The results obtained indicate a clear need: to protect and enhance the first generation of *A. mali*. The use of forecasting tools, such as the degree-day model of Bangels et al. (2021), to predict both the first *A. mali* flight and the start of *E. lanigerum* activity can be very useful from an IPM perspective. Overall, our results provide a framework for incorporating parasitoid monitoring and infestation thresholds into practical decision-support systems. From a practical standpoint, infestation levels below the estimated breakpoint (∼ 4.81) should not automatically trigger chemical interventions, as parasitoid populations may still be increasing. As infestation approaches the threshold, monitoring should be intensified, paying attention to adult parasitoid abundance and parasitism rates. When *E. lanigerum* infestations exceed the threshold, intervention decisions should consider both aphid density and parasitoid activity, rather than relying solely on the density-based threshold. Furthermore, applying this threshold to other production areas should be undertaken cautiously, as climatic conditions, cultivar susceptibility, and rootstock characteristics may influence the infestation level at which effective regulation occurs. Local validation would therefore be advisable. Improving the synchrony between early aphid build-up and parasitoid appearance, through selective pesticide use and conservation of early parasitoid generations, remains essential for maintaining effective biological control and supporting the transition toward pesticide-reduction policies in European apple production.

## Supplementary Material

toag084_Supplementary_Data
