# Supplementary material for: Seasonal abundance of Eriosoma lanigerum (Hausmann) (Hemiptera: Aphididae) and its regulation by Aphelinus mali (Haldeman) (Hymenoptera: Aphelinidae) from a 3-year survey in apple orchards
Source: J Econ Entomol. 2026 Apr 7;119(4):2476–86. doi: 10.1093/jee/toag084 (PMC13462274; doi:10.1093/jee/toag084)
Supplement: Supplementary file 1 [file toag084_supplementary_data.docx]

Additional characteristics of the surveyed orchards and treatments carried out

| Orchard | Year of planting | Cultivar | Rootstock | 2021 | 2022 | 2023 |
| --- | --- | --- | --- | --- | --- | --- |
| AO1 | 2015 | Gala | M9T337 | azadirachtin (Apr 5, May 7),  potassium salts of fatty acids (fortnight during the summer) | water washing on *E. lanigerum* colonies (Jul 16) | None |
| AO2 | 2001 | Golden Delicious | Ungrafted | azadirachtin (Mar 31, Apr 23, May 01),  potassium salts of fatty acids (Jul 10, Jul 25) | water washing on *E. lanigerum* colonies (Jul 17) | pirimicarb (Mar 02), sulfoxaflor (May 03) |
| AO3 | 2012 | Gala | M9T337 | flonicamid (Mar 26), sulfoxaflor (Apr 29), chlorpyrifos-methyl (Jun 24) | flonicamid (Apr 04), sulfoxaflor (May 02), pirimicarb (Jun 20) | pyriproxyfen (Mar 24), primicarb (Mar 30), sulfoxaflor (May 08) |
| AO4 | 2003 | Gala | M9T337 |  | horticultural oil (Mar 4, Mar 11), azadirachtin (Apr 5, Apr 27) | horticultural oil (Mar 2, Mar 16), azadirachtin (Apr 18) |
| AO5 | 2007 | Gala | M9T337 |  | azadirachtin (Apr 07, Apr 26, Jun 04), potassium salts of fatty acids (May 27, May 29) | azadirachtin (Apr 2, Apr 18, Jul 26), potassium salts of fatty acids (May 15, May 27) |
